# Electrical Impedance Monitoring of C2C12 Myoblast Differentiation on an Indium Tin Oxide Electrode

**DOI:** 10.3390/s16122068

**Published:** 2016-12-05

**Authors:** Ilhwan Park, Yeonhee Hong, Young-Hoo Jun, Ga-Yeon Lee, Hee-Sook Jun, Jae-Chul Pyun, Jeong-Woo Choi, Sungbo Cho

**Affiliations:** 1 Department of Biomedical Engineering, Gachon University, Incheon 21936, Korea; orange1537@gmail.com; 2 College of Pharmacy and Gachon Institute of Pharmaceutical Science, Gachon University, Incheon 21936, Korea; hyhbona@naver.com (Y.H.); hsjun@gachon.ac.kr (H.-S.J.); 3 Lee Gil Ya Cancer and Diabetes Institute, Incheon 21999, Korea; yhjun17@pupils.nlcsjeju.kr; 4 Department of Materials Science and Engineering, Yonsei University, Seodaemun-gu, Seoul 120-749, Korea; gayeon@yonsei.ac.kr (G.-Y.L.); jcpyun@yonsei.ac.kr (J.-C.P.); 5 Gachon Advanced Institute for Health Science & Technology, Gachon University, Incheon 21999, Korea; 6 Department of Chemical and Biomolecular Engineering, Sogang University, Seoul 04107, Korea; jwchoi@sogang.ac.kr

**Keywords:** electric cell-substrate impedance sensing, label-free, real-time, C2C12 cells, myoblast differentiation

## Abstract

Electrical cell-substrate impedance sensing is increasingly being used for label-free and real-time monitoring of changes in cell morphology and number during cell growth, drug screening, and differentiation. In this study, we evaluated the feasibility of using ECIS to monitor C2C12 myoblast differentiation using a fabricated indium tin oxide (ITO) electrode-based chip. C2C12 myoblast differentiation on the ITO electrode was validated based on decreases in the mRNA level of MyoD and increases in the mRNA levels of myogenin and myosin heavy chain (MHC). Additionally, MHC expression and morphological changes in myoblasts differentiated on the ITO electrode were comparable to those in cells in the control culture dish. From the monitoring the integration of the resistance change at 21.5 kHz, the cell differentiation was label-free and real-time detectable in 30 h of differentiation (*p* < 0.05).

## 1. Introduction

Skeletal muscle, the largest organ of the body, constitutes 40%–50% of the total body mass and plays an important role in movement, insulation of internal organs, and metabolism [1,2]. Impairment of muscle function can lead to many pathological changes including diabetes, obesity, and muscle wasting, as well as aging [3,4,5]. In vitro studies using muscle cells have been carried out as a means of predicting in vivo effects. However, the reproducible production of sufficient quantities of well-defined differentiated cells is one of the major hurdles in such studies. In addition, new technologies to quantitatively evaluate the functionality of cells are required to obtain consistent results. The C2C12 cell line is a mouse myoblast cell line that expresses various proteins during differentiation into myotubes (myogenesis). C2C12 myoblast and differentiated C2C12 cells have been used for various in vitro studies, including studies on muscle cell regeneration, metabolism, insulin actions, and muscle atrophy [6,7,8], in association with mechanistic studies of diseases such as diabetes, chronic heart failure, and chronic kidney disease [9].

To accurately study cellular responses or behaviors to external stimuli, real-time and non-destructive measurements that characterize physiological and morphological changes in cells need to be employed. Electrical cell-substrate impedance sensing (ECIS) developed by Giaver and Keese is one method for real-time and label-free detection of cellular behaviors [10,11]. The frequency-dependent electrical impedance of cells covering an electrode is achieved from the in-phase and out of phase potentials measured while applying a weak alternating electric field [12]. The values of cell parameters can be extracted using a fitting analysis with equivalent circuit models designed to describe the current path and the potential distribution on the interface between the electrode and the cells [13,14]. ECIS allows for high-throughput and non-destructive screening of cellular responses to drug candidates or culture conditions [15,16]. It has been used to quantify cell adhesion [17,18], proliferation [19,20], metastasis [21], necrosis [22], apoptosis [23,24], wound healing [25,26], and differentiation [27,28]. We previously reported the electrical impedance characterization of human mesenchymal stem cell (hMSC) growth [29], hMSC differentiation into adipocytes [30], osteogenic differentiation of hMSCs [31], neural differentiation of hMSCs [32], adipose tissue-derived stem cell (ADSC) growth [33], and senescence of ADSCs [34]. Additionally, the effect of the electrode material and structure patterned by nanoparticles [35], graphene [36], or a mixture of nanoparticles and graphene [37] on stem cell differentiation was investigated. Our experiments in which we measured the electrochemical signals of differentiated or undifferentiated stem cells showed that the electrochemical signature can be used to quantify the pluripotency of the stem cells [38,39].

In this research, we evaluated the feasibility of a transparent indium tin oxide (ITO) electrode for ECIS of the changes in C2C12 cell density on the electrode during myoblast differentiation. The change in the cell thickness according to the myotube formation on the gold electrode was measured by ECIS to characterize the myotube atrophy and hypertrophy with respect to various stimuli [40,41]. To minimize the impedance variation caused by the different cell adhesion on the electrode surface, an extracellular matrix gel was coated on the electrode. Previous studies reported the feasibility of the ITO electrode to characterize the coating of protein layers and the cell-protein interactions [42,43]. Here, the myoblast differentiation of C2C12 cells on the gel-coated ITO electrode was demonstrated by analyzing the mRNA level of myogenic factors (MyoD, myogenin, and myosin heavy chain (MHC)). Additionally, morphological changes and MHC expression in myoblasts differentiated on the ITO electrode were compared to those in cells grown in a control culture dish. Finally, the impedance monitoring of C2C12 myoblast differentiation on the ITO electrode was evaluated.

## 2. Materials and Methods

### 2.1. Fabrication of ITO Electrode-Based Chip

**Figure 1:**
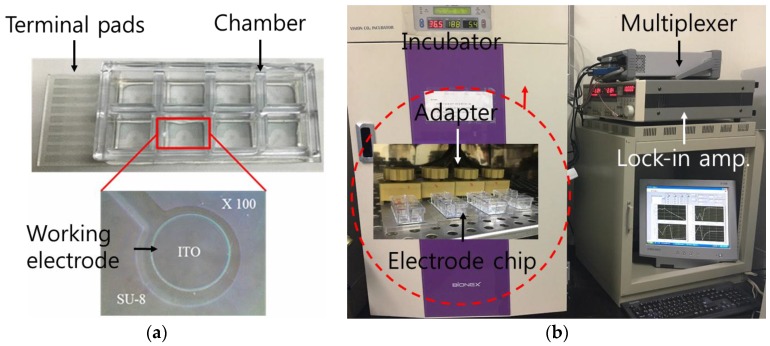
(**a**) Fabricated indium tin oxide (ITO) electrode chip consisting of chamber and terminal pads; (**b**) experimental setup for the impedance measurement of cells.

For the ITO electrode, the chip established in previous studies [42,43,44] was used. An ITO electrode with a thickness of 480 nm was patterned on slide glass (75 mm × 25 mm × 1.1 mm), and consisted of eight working electrodes, a common counter electrode, transmission lines, and terminal pads. Using spin coating and photolithography, an epoxy-based photoresist (SU-8 2002, Microchem, Newton, MA, USA) was used to provide a 2-μm-thick coating to insulate the transmission lines of the ITO electrode chip. The exposed working electrodes (WE) were disk-shaped with a radius of 250 μm and separated from the counter electrode (CE) by a distance of 3 mm. A polystyrene chamber was attached on the ITO electrode-based chip using adhesive silicone to provide a reservoir to contain the culture medium for the cells (Figure 1a).

### 2.2. C2C12 Myoblast Cultivation and Differentiation

To achieve stable cell adhesion on the ITO electrode, the surface of the electrode substrate was covered with 100 μL of Matrigel (0.9–1.2 mg·mL^−1^, BD MatrigelTM Matrix, BD Biosciences, San Jose, CA, USA). The thickness of the Matrigel layer coated on the ITO electrode was measured using atomic force microscopy (AFM, XE-100, Parks Systems, Suwon, Korea). C2C12 myoblasts (105 cells) were seeded on the Matrigel-coated ITO electrode and cultured in Dulbecco’s modified Eagle’s medium (DMEM) supplemented with 10% fetal bovine serum (FBS, Welgene, Daegu, Korea), 100 IU·mL^−1^ penicillin, 0.1 mg·mL^−1^ streptomycin in 5% CO_2_ at 37 °C. To induce myoblast differentiation, C2C12 differentiation medium containing DMEM, 2% horse serum (HS, Gibco, Carlsbad, CA, USA), 100 IU·mL^−1^ penicillin, and 0.1 mg·mL^−1^ streptomycin was applied. For the control group, the culture medium was switched to a maintenance medium (DMEM, 0.5% FBS, 1% penicillin) to suppress cell proliferation and avoid the delamination of the cell layer resulting from the excessive growth of cells. The number of cells cultured on the sensing electrode with or without the differentiation medium was counted using the ImageJ software (NIH, Bethesda, MD, USA).

### 2.3. Electrical Impedance Measurement of Cells

During cell culture and differentiation, electrical impedance spectra were measured in the frequency range of 100 Hz to 100 kHz using a digital lock-in amplifier with a built-in voltage generator (SR830, Stanford Research Systems, Sunnyvale, CA, USA) and multiplexer (34970A, Agilent Technologies, Inc., Santa Clara, CA, USA) [45,46]. Figure 1b shows a photo of an experimental setup for the impedance measurement of cells. The amplitude of the flowing current was limited to below 1 μA by connecting the resistor in series with the voltage generator to avoid the effect of the electrical field on the C2C12 cells [47]. The instruments used for ECIS were controlled by the LabVIEW program (National Instruments, Austin, TX, USA) through general purpose interface bus communication. The impedance data were calculated from the applied and measured potentials.

### 2.4. RNA Isolation and Quantitative Real-Time PCR

Total RNA was isolated from cells using Trizol reagent according to the manufacturer’s protocol (RNAiso Plus, TaKaRa, Dalian, China). RNA quantity was measured using an ND-1000 spectrophotometer (Thermo Scientific, Waltham, MA, USA). After cDNA was synthesized from 2 μg of RNA using the PrimeScript 1st strand cDNA synthesis kit (TaKaRa), quantitative real-time PCR (qRT-PCR) was performed in a reaction mixture containing SYBR Green master mix (TaKaRa). PCR amplification was carried out using the CFX384 Touch™ Real-Time PCR Detection System (Bio-Rad, Munich, Germany) and stopped at 40 cycles. The relative gene expression levels were normalized according to the cycle threshold method. The sequences of the primer pairs used were as follows: Cyclophilin forward: 5′-TGGAGAGCACCAAGACAGACA-3′, reverse: 5′-TGCCGGAGTCGACAATGAT-3′, MyoD forward: 5′-CTTCTATCGCCGCCACTC-3′, reverse: 5′-AAGTCGTCTGCTGTCTCAA-3′, myogenin forward: 5′-CCAACCCAGGAGATCATTTG-3′, reverse: 5′-ACGATGGACGTAAGGGAGTG-3′, and MHC forward: 5′-ACAAGCTGCGGGTGAAGAGC-3′, reverse: 5′-CAGGACA GTGACAAAGAACG-3′.

### 2.5. Immunofluorescence Analysis

Cells were fixed in 4% paraformaldehyde for 20 min at room temperature and washed with PBS. Cells were then permeabilized at room temperature with PBS containing 0.27% Triton X-100 and incubated with protein blocking solution (DAKO). The cells were exposed to anti-MHC antibody overnight at 4 °C and then incubated with fluorescein isothiocyanate (FITC)-conjugated secondary antibody followed by DAPI staining. Fluorescence images of the cells were taken using a confocal microscope.

### 2.6. Statistical Analysis

All values were expressed as mean ± SD. Statistical analyses were performed by IBM SPSS statistics 20. Statistical significance was defined as a value of *p* < 0.05.

## 3. Results and Discussion

**Figure 2:**
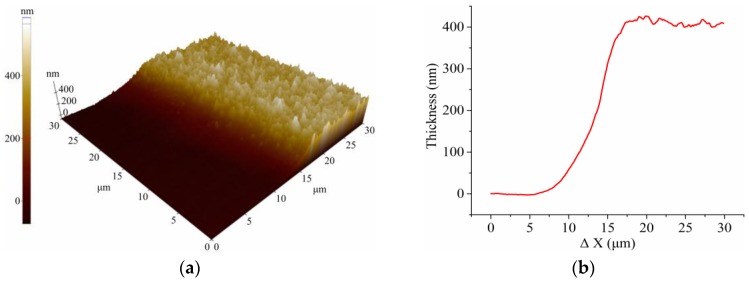

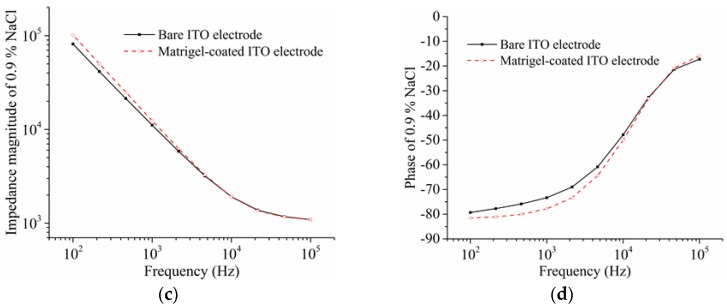
(**a**) AFM image and (**b**) height data of the extracellular matrix gel coated on the ITO electrode; (**c**) impedance magnitude and (**d**) phase of the bare or gel-coated ITO electrode in 0.9% NaCl electrolyte.

The extracellular matrix gel was uniformly coated on the ITO electrode surface with a thickness of approximately 410 nm. Figure 2a,b shows an AFM image of the surface topology of the gel coated on the ITO and the thickness plot, respectively. The impedance spectra of the bare or the gel-coated ITO electrode measured in 0.9% NaCl electrolyte were shown in Figure 2c,d. The impedance characteristic of the fabricated electrode was determined by the electrode interfacial impedance at low frequencies and the solution resistance at high frequencies. The gel coating on the electrode led to a slight increase in the reactance at low frequencies, resulting in the increase of the impedance magnitude and the decrease of the phase. This could be derived from an additional capacitance characteristic of the coating layer in series with the capacitive electrode impedance.

**Figure 3:**
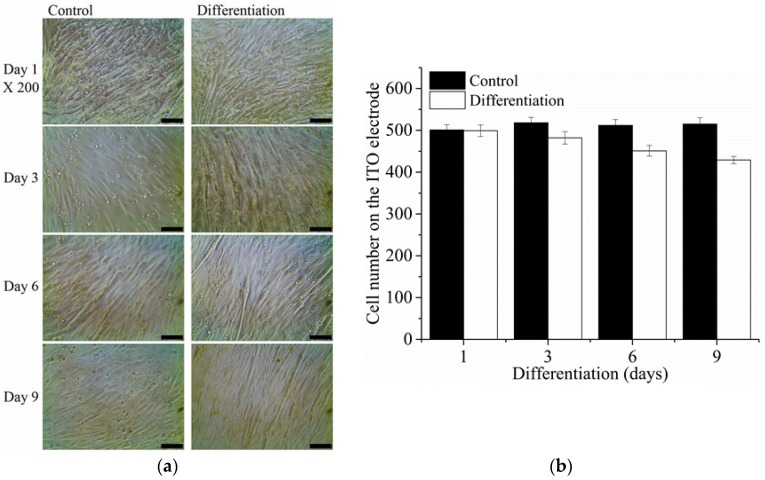
(**a**) Phase contrast micrograph (scale bar: 100 μm) and (**b**) counted number of C2C12 cells cultured on the extracellular matrix gel-coated ITO working electrode with or without differentiation medium.

During culture in growth medium with 10% FBS, the C2C12 cells adhered, spread, and proliferated on the gel-coated ITO electrodes (Figure 3a). After replacing the growth medium with differentiation medium containing 2% HS after 3 days of culture, the myotubular morphology of the cells was observed at 7 or 11 days of culture. On the other hand, cells in the control group did not show any morphological changes relevant to differentiation into myotubes. The myogenic differentiation affected the cell proliferation as shown in Figure 3b. The number of cells on the sensing electrode with the differentiation medium was lower than the control at 7 or 11 days of culture.

MyoD is expressed in myoblasts and decreases during differentiation, while myogenin and MHC are expressed in a later stage of myogenesis [48,49]. By analyzing the expression of the myogenic factors involved in myogenesis, it was found that the mRNA levels of MyoD expressed in myoblasts differentiated both on the cell culture dish and the ITO electrode decreased during differentiation.

**Figure 4:**
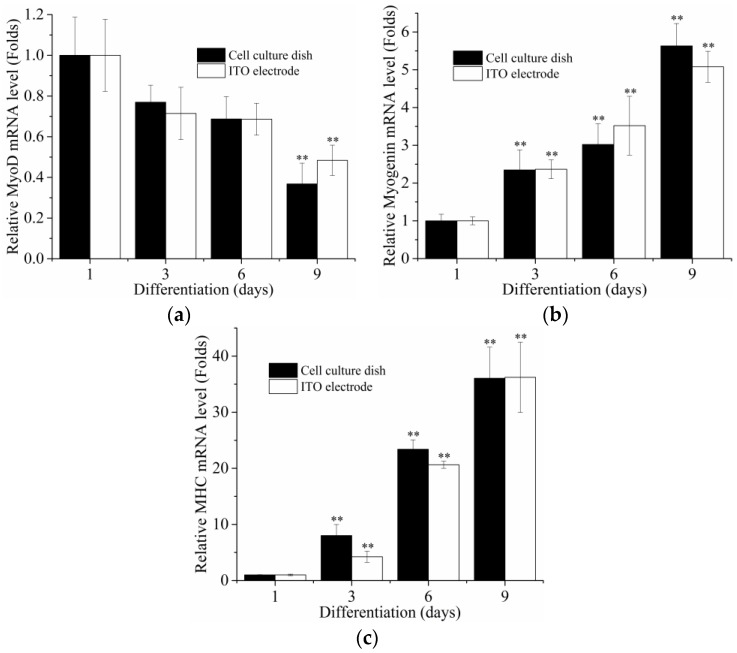
Expression of mRNA for (**a**) MyoD; (**b**) Myogenin; and (**c**) myosin heavy chain (MHC) was analyzed by qRT-PCR during differentiation (Days 1, 3, 6 and 9) on a cell culture dish or on the ITO electrode (*n* = 3). Relative expression was normalized to cyclophilin expression. ** *p* < 0.01 compared with Day 1.

**Figure 5:**
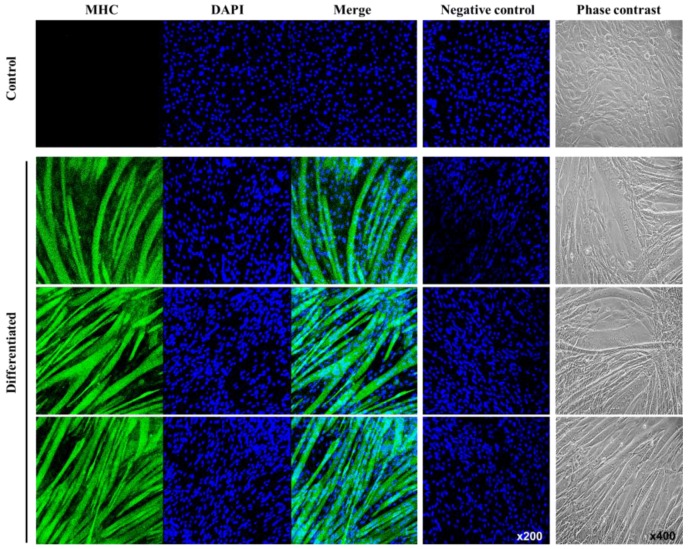
Changes in the morphology and MHC expression in myotubes differentiated from C2C12 myoblasts. MHC expression was detected by staining with an anti-MHC antibody (**Green**), while nuclei were detected by DAPI staining (**Blue**).

Additionally, the levels of myogenin and MHC mRNA in cells both on the cell culture dish and on the ITO electrode increased during differentiation (Figure 4). In our immunofluorescence analysis, we observed the multinuclear myotube and the increased MHC expression (green) in differentiated C2C12 cells, whereas undifferentiated C2C12 cells (control) exhibited only nuclei (blue) (Figure 5). Our results indicated that C2C12 cells were successfully differentiated into myotubes on the ITO electrode using our differentiation protocol.

**Figure 6:**
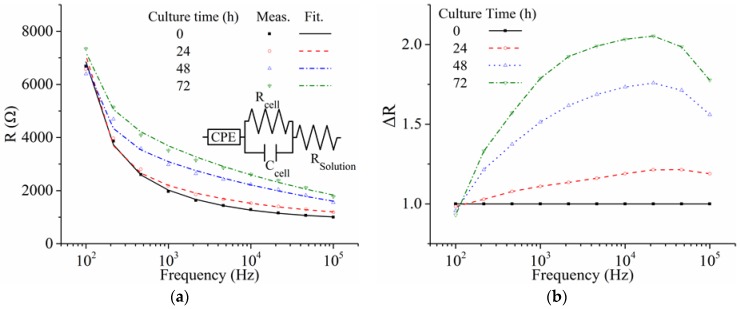
(**a**) Resistance (R) of C2C12 cells on the ITO electrode measured during cell growth, together with fitted lines using the established equivalent circuit model [34] and (**b**) resistance change (ΔR = R/R at 0 h).

Figure 6a shows the electrical impedance spectra of the resistance (R) of the C2C12 myoblasts on the ITO electrode measured during cell growth, together with the fitted lines using the established equivalent circuit model [34]. The electrical impedance characteristics of the cells on the electrode were well represented in the frequency range of 100 Hz to 100 kHz by the sum of a constant phase element for the electrode interfacial impedance (CPE), a parallel resistor (R_cell_) and capacitor (C_cell_) for the impedance of cells, and a solution resistor (R_solution_). In Figure 6b, indicating resistance change (ΔR = R/R at 0 h), the value was most significantly changed at a frequency of 21.5 kHz during cell growth.

**Figure 7:**
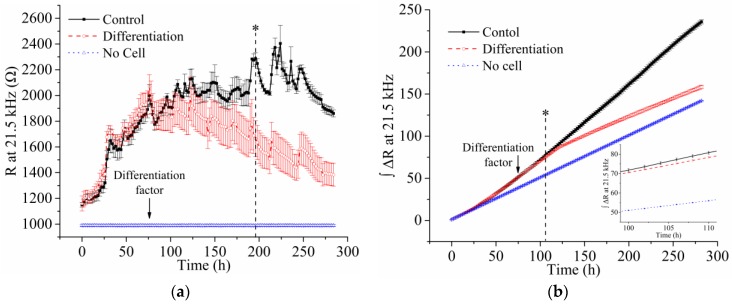
(**a**) Resistance (R) at 21.5 kHz and (**b**) integration of the resistance change (ΔR) at 21.5 kHz monitored during cell growth or differentiation. The average and standard error of the measured data (*n* = 4) are represented by the symbol and bar, respectively.

Figure 7a shows the average (symbol) and standard error (bar) of the resistance at 21.5 kHz (*n* = 4) monitored during cell cultivation with or without the differentiation medium. The arrows in the figure indicate the time at which the culture medium was replaced with either differentiation or maintenance medium. All media were refreshed every day during the impedance monitoring of the cells. Before applying the differentiation medium, the resistance increased with fluctuations due to cell adhesion and spreading on the ITO electrode. However, those values gradually decreased during myoblast differentiation and were distinguishable from the values of the control group by 116 h of differentiation time (*p*-value of the Student’s *t*-test < 0.05). The decrease in the value during differentiation was caused by the decrease in cell density on the electrode together with changes in cell morphology. Figure 7b shows the integration of the resistance change at 21.5 kHz of C2C12 cells on the ITO electrode during cell growth or differentiation. The integration of the value resulted in not only the reduction of the deviation between the measured values but also faster detection of the cell differentiation. According to the integration of the resistance change at 21.5 kHz, the cell differentiation was distinguishable from the control group in 30 h of differentiation time (*p*-value of the Student’s *t*-test < 0.05).

Our experimental results demonstrated a feasibility of the ITO electrode and electrical impedance monitoring to characterize C2C12 myoblast differentiation, which resulted in the changes in cell morphology and density. The suggested differentiation protocol and experimental conditions were suitable for label-free and real-time monitoring of myoblast differentiation on the ITO electrode. Thus, it is expected that this differentiated cell-based chip can be utilized for the development of therapy or drug candidates for muscle diseases.

## 4. Conclusions

We evaluated the feasibility of using an ITO electrode for label-free and real-time characterization of C2C12 myoblast differentiation using electrical impedance analysis. The C2C12 myoblasts adhered well, proliferated, and differentiated on the extracellular matrix gel-coated ITO electrode. Furthermore, differentiation of C2C12 cells on the electrode was validated by analyzing myogenic gene expressions, changes in cell morphology, and immunofluorescence comparable to cells on a culture dish. The electrical impedance characteristics of the C2C12 cells on the gel-coated ITO electrode could be explained by the established equivalent circuit modeling. During myogenic differentiation, the integration of the resistance change at 21.5 kHz was separated from the values in the control group at 30 h of differentiation time. Our experimental result demonstrated the feasibility of using the ITO electrode for real-time and label-free characterization of C2C12 myoblast differentiation.

## References

[1] Lindstedt S.L. (2016). Skeletal muscle tissue in movement and health: Positives and negatives. J. Exp. Biol..

[2] Mayeuf-Louchart A., Staels B., Duez H. (2015). Skeletal muscle functions around the clock. Diabetes Obes. Metab..

[3] Russell A.P., Foletta V.C., Snow R.J., Wadley G.D. (2014). Skeletal muscle mitochondria: A major player in exercise, health and disease. BBA Gen. Subj..

[4] Ryall J.G. (2012). The role of sirtuins in the regulation of metabolic homeostasis in skeletal muscle. Curr. Opin. Clin. Nutr..

[5] Kinugawa S., Takada S., Matsushima S., Okita K., Tsutsui H. (2015). Skeletal Muscle Abnormalities in Heart Failure. Int. Heart J..

[6] D’Andrea P., Scaini D., Severino L.U., Borelli V., Passamonti S., Lorenzon P., Bandiera A. (2015). In vitro myogenesis induced by human recombinant elastin-like proteins. Biomaterials.

[7] Litwiniuk A., Pijet B., Pijet-Kucicka M., Gajewska M., Pajak B., Orzechowski A. (2016). FOXO1 and GSK-3 beta Are Main Targets of Insulin-Mediated Myogenesis in C2C12 Muscle Cells. PLoS ONE.

[8] Rebbapragada A., Benchabane H., Wrana J.L., Celeste A.J., Attisano L. (2003). Myostatin signals through a transforming growth factor beta-like signaling pathway to block adipogenesis. Mol. Cell. Biol..

[9] D’Souza D.M., Al-Sajee D., Hawke T.J. (2013). Diabetic rnyopathy: Impact of diabetes mellitus on skeletal muscle progenitor cells. Front. Physiol..

[10] Giaever I., Keese C.R. (1984). Monitoring fibroblast behavior in tissue culture with an applied electric field. Proc. Natl. Acad. Sci. USA.

[11] Giaever I., Keese C.R. (1991). Micromotion of mammalian cells measured electrically. Proc. Natl. Acad. Sci. USA.

[12] Wegener J., Keese C.R., Giaever I. (2000). Electric cell–substrate impedance sensing (ECIS) as a noninvasive means to monitor the kinetics of cell spreading to artificial surfaces. Exp. Cell Res..

[13] Wang L., Wang H., Wang L., Mitchelson K., Yu Z.Y., Cheng J. (2008). Analysis of the sensitivity and frequency characteristics of coplanar electrical cell–substrate impedance sensors. Biosens. Bioelectron..

[14] Silva B.P.G., de Florio D.Z., Brochsztain S. (2014). Characterization of a Perylenediimide Self-Assembled Monolayer on Indium Tin Oxide Electrodes Using Electrochemical Impedance Spectroscopy. J. Phys. Chem. C.

[15] Ceriotti L., Ponti J., Broggi F., Kob A., Drechsler S., Thedinga E., Colpo P., Sabbioni E., Ehret R., Rossi F. (2007). Real-time assessment of cytotoxicity by impedance measurement on a 96-well plate. Sens. Actuators B Chem..

[16] Daza P., Olmo A., Canete D., Yufera A. (2013). Monitoring living cell assays with bio-impedance sensors. Sens. Actuators B Chem..

[17] Chen S.W., Yang J.M., Yang J.H., Yang S.J., Wang J.S. (2012). A computational modeling and analysis in cell biological dynamics using electric cell–substrate impedance sensing (ECIS). Biosens. Bioelectron..

[18] Qiu Y.L., Liao R.L., Zhang X. (2008). Real-time monitoring primary cardiomyocyte adhesion based on electrochemical impedance spectroscopy and electrical cell–substrate impedance sensing. Anal. Chem..

[19] Xiao C., Lachance B., Sunahara G., Luong J.H.T. (2002). An in-depth analysis of electric cell–substrate impedance sensing to study the attachment and spreading mammalian cells. Anal. Chem..

[20] Liu Q.J., Yu J.J., Xiao L., Tang J.C.O., Zhang Y., Wang P., Yang M. (2009). Impedance studies of bio-behavior and chemosensitivity of cancer cells by micro-electrode arrays. Biosens. Bioelectron..

[21] Nguyen T.A., Yin T.I., Reyes D., Urban G.A. (2013). Microfluidic Chip with Integrated Electrical Cell-Impedance Sensing for Monitoring Single Cancer Cell Migration in Three-Dimensional Matrixes. Anal. Chem..

[22] Qiu Y.L., Liao R.L., Zhang X. (2009). Impedance-Based Monitoring of Ongoing Cardiomyocyte Death Induced by Tumor Necrosis Factor-alpha. Biophys. J..

[23] Arndt S., Seebach J., Psathaki K., Galla H.J., Wegener J. (2004). Bioelectrical impedance assay to monitor changes in cell shape during apoptosis. Biosens. Bioelectron..

[24] Yin H.Y., Wang F.L., Wang A.L., Cheng J., Zhou Y.X. (2007). Bioelectrical impedance assay to monitor changes in aspirin-treated human colon cancer HT-29 cell shape during apoptosis. Anal. Lett..

[25] Keese C.R., Wegener J., Walker S.R., Giaever L. (2004). Electrical wound-healing assay for cells in vitro. Proc. Natl. Acad. Sci. USA.

[26] Yang J.M., Chen S.W., Yang J.H., Hsu C.C., Wang J.S. (2016). A quantitative cell modeling and wound-healing analysis based on the Electric Cell–substrate Impedance Sensing (ECIS) method. Comput. Biol. Med..

[27] Kramer A.H., Joos-Vandewalle J., Edkins A.L., Frost C.L., Prinsloo E. (2014). Real-time monitoring of 3T3-L1 preadipocyte differentiation using a commercially available electric cell–substrate impedance sensor system. Biochem. Biophys. Res. Commun..

[28] Bagnaninchi P.O., Drummond N. (2011). Real-time label-free monitoring of adipose-derived stem cell differentiation with electric cell–substrate impedance sensing. Proc. Natl. Acad. Sci. USA.

[29] Cho S.B., Thielecke H. (2008). Electrical characterization of human mesenchymal stem cell growth on microelectrode. Microelectron. Eng..

[30] Cho S., Gorjup E., Thielecke H. (2009). Chip-based time-continuous monitoring of toxic effects on stem cell differentiation. Ann. Anat..

[31] Hildebrandt C., Buth H., Cho S.B., Impidjati, Thielecke H. (2010). Detection of the osteogenic differentiation of mesenchymal stem cells in 2D and 3D cultures by electrochemical impedance spectroscopy. J. Biotechnol..

[32] Park H.E., Kim D., Koh H.S., Cho S., Sung J.S., Kim J.Y. (2011). Real-Time Monitoring of Neural Differentiation of Human Mesenchymal Stem Cells by Electric Cell–substrate Impedance Sensing. J. Biomed. Biotechnol..

[33] Jun H.S., Choi W., Kim J.Y., Cho S. (2013). Electrical Impedance Characterization of Adipose Tissue-Derived Stem Cells Cultured on Indium Tin Oxide Electrodes. J. Biomed. Nanotechnol..

[34] Jun H.S., Dao L.T.M., Pyun J.C., Cho S. (2013). Effect of cell senescence on the impedance measurement of adipose tissue-derived stem cells. Enzyme Microb. Technol..

[35] Kim T.H., Yea C.H., Chueng S.T.D., Yin P.T.T., Conley B., Dardir K., Pak Y., Jung G.Y., Choi J.W., Lee K.B. (2015). Large-Scale Nanoelectrode Arrays to Monitor the Dopaminergic Differentiation of Human Neural Stem Cells. Adv. Mater..

[36] Kim T.H., Shah S., Yang L.T., Yin P.T., Hossain M.K., Conley B., Choi J.W., Lee K.B. (2015). Controlling Differentiation of Adipose-Derived Stem Cells Using Combinatorial Graphene Hybrid-Pattern Arrays. ACS Nano.

[37] Kim T.H., Lee K.B., Choi J.W. (2013). 3D graphene oxide-encapsulated gold nanoparticles to detect neural stem cell differentiation. Biomaterials.

[38] Yea C.H., Jeong H.C., Moon S.H., Lee M.O., Kim K.J., Choi J.W., Cha H.J. (2016). In situ label-free quantification of human pluripotent stem cells with electrochemical potential. Biomaterials.

[39] Yea C.H., An J.H., Kim J., Choi J.W. (2013). In situ electrochemical detection of embryonic stem cell differentiation. J. Biotechnol..

[40] Rakhilin S., Turner G., Katz M., Warden R., Irelan J., Abassi Y.A., Glass D.J. (2011). Electrical Impedance as a Novel Biomarker of Myotube Atrophy and Hypertrophy. J. Biomol. Screen..

[41] Murphy S.M., Kiely M., Jakeman P.M., Kiely P.A., Carson B.P. (2016). Optimization of an in vitro bioassay to monitor growth and formation of myotubes in real time. Biosci. Rep..

[42] Cho S. (2012). Electrical Impedance Simulation and Characterization of Cell Growth Using the Fricke Model. J. Nanosci. Nanotechnol..

[43] Choi Y., Yagati A.K., Cho S. (2015). Electrochemical Characterization of Poly-L-Lysine Coating on Indium Tin Oxide Electrode for Enhancing Cell Adhesion. J. Nanosci. Nanotechnol..

[44] Choi Y.H., Min J., Cho S. (2015). Indium tin oxide based chip for optical and electrochemical characterization of protein-cell interaction. Jpn. J. Appl. Phys..

[45] Lee G.H., Pyun J.C., Cho S. (2014). Electrical Impedance Characterization of Cell Growth on Interdigitated Microelectrode Array. J. Nanosci. Nanotechnol..

[46] Park J., Hwang K.S., Cho S. (2015). Dependence of Impedance Measurement Sensitivity of Cell Growth on Sensing Area of Circular Interdigitated Electrode. J. Nanosci. Nanotechnol..

[47] Aas V., Torbla S., Andersen M.H., Jensen J., Rustan A.C. (2002). Electrical stimulation improves insulin responses in a human skeletal muscle cell model of hyperglycemia. Ann. N. Y. Acad. Sci..

[48] Lee S.J., Yoo M., Go G.Y., Kim D.H., Choi H., Leem Y.E., Kim Y.K., Seo D.W., Ryu J.H., Kang J.S. (2016). Bakuchiol augments MyoD activation leading to enhanced myoblast differentiation. Chem. Biol. Interact..

[49] Wang M., Yu H., Kim Y.S., Bidwell C.A., Kuang S.H. (2012). Myostatin facilitates slow and inhibits fast myosin heavy chain expression during myogenic differentiation. Biochem. Biophys. Res. Commun..

